# Loss of control eating in children is associated with altered cortical and subcortical brain structure

**DOI:** 10.3389/fpsyg.2023.1237591

**Published:** 2024-01-11

**Authors:** Alaina L. Pearce, Bari Fuchs, Shana Adise, Travis D. Masterson, Nicole Fearnbach, Laural English, Kathleen L. Keller

**Affiliations:** ^1^Department of Nutritional Science, The Pennsylvania State University, University Park, PA, United States; ^2^Division of Endocrinology, Diabetes, and Metabolism, Children's Hospital Los Angeles, Los Angeles, CA, United States; ^3^Department of Health and Life Sciences, Florida State University, Tallahassee, FL, United States; ^4^United States Department of Agriculture, Washington, DC, United States; ^5^Department of Food Science, The Pennsylvania State University, University Park, PA, United States

**Keywords:** loss of control-eating, gray matter volume, surface morphology, children, MRI

## Abstract

**Introduction:**

Loss of control (LOC) eating is the perceived inability to control how much is eaten, regardless of actual amount consumed. Childhood LOC-eating is a risk factor for the development of binge-eating disorder (BED), but its neurobiological basis is poorly understood. Studies in children with BED have shown both increased gray matter volume in regions related to top-down cognitive control (e.g., dorsolateral prefrontal cortex) and reward-related decision making (e.g., orbital frontal cortex) relative to healthy controls. However, no studies have examined brain structure in children with LOC-eating. To identify potential neurobiological precursors of BED, we conducted secondary analysis of five studies that conducted T1 MPRAGE scans.

**Methods:**

A total of 143, 7–12-year-old children (*M* = 8.9 years, 70 boys) were included in the study, 26% of which (*n* = 37) reported LOC-eating (semi-structured interview). Age, sex, and obesity status did not differ by LOC-eating. Differences between children with and without LOC were examined for gray matter volume, cortical thickness, gyrification, sulci depth, and cortical complexity after adjusting for age, sex, total intercranial volume, weight status, and study.

**Results:**

Children with LOC, relative to those without, had greater gray matter volume in right orbital frontal cortex but lower gray matter volume in right parahippocampal gyrus, left CA4/dentate gyrus, and left cerebellar lobule VI. While there were no differences in cortical thickness or gyrification, children with LOC-eating had great sulci depth in left anterior cingulate cortex and cuneus and greater cortical complexity in right insular cortex.

**Discussion:**

Together, this indicates that children with LOC-eating have structural differences in regions related to cognitive control, reward-related decision-making, and regulation of eating behaviors.

## 1 Introduction

One in four children report experiencing loss of control (LOC)-eating, which is the perceived inability to stop eating, regardless of the amount consumed (Tanofsky-Kraff et al., 2020). LOC-eating was first conceptualized as a way to capture eating behaviors that precede the development of binge eating disorder (BED; Tanofsky-Kraff et al., 2007) and it is an important risk factor for the development of BED in early adolescence (Tanofsky-Kraff et al., 2011; Hilbert et al., 2013). In children, LOC-eating increases risk for adverse outcomes such as greater adiposity (Shomaker et al., 2010) and obesity (Tanofsky-Kraff et al., 2009b). However, independent of child weight status, LOC-eating has been associated with metabolic dysfunction (Tanofsky-Kraff et al., 2012; Byrne et al., 2019) and systemic inflammation (Shank et al., 2016). LOC-eating in adolescence has also been associated with disordered eating attitudes, BED, depression, and anxiety, and again, these relationships are independent of the effects body weight (Shomaker et al., 2010; Byrne et al., 2019; Tanofsky-Kraff et al., 2020). Given the broad impacts of LOC-eating across development, it is critical to clarify the neurobiological systems that are associated with this behavior. Therefore, this study examined differences in gray matter volume and surface morphology between children who reported LOC-eating and those that did not.

To our knowledge, there have been no prior studies examining whether LOC-eating is associated with alterations in brain structure. In general, higher transdiagnostic levels of psychopathology have been associated with lower global gray matter volume and surface area in children (Mewton et al., 2022; Bashford-Largo et al., 2023; Romer et al., 2023). More closely related to LOC-eating, there is initial evidence for structural differences associated with BED. Among children with healthy weight, higher levels of binge eating have been associated with greater gray matter volume in the insula (Chen et al., 2022), a region implicated in gustation, visceral interoception, and reward processing (Frank et al., 2013; Uddin et al., 2017; Centanni et al., 2021). Recent evidence from the Adolescent Brain Cognitive Development (ABCD) ® study also found that children with BED had greater gray matter density in regions that support top-down cognitive control (e.g., dorsolateral prefrontal cortex—dlPFC, middle frontal gyrus, superior frontal gyrus) and reward-related decision making (anterior cingulate, orbital frontal cortex—OFC) compared to body mass index (BMI) matched controls (Murray et al., 2022). Similarly, adolescents with co-morbid obesity and BED have greater gray matter volume in OFC compared to adolescents without BED, regardless of weight status (Turan et al., 2021). Therefore, initial evidence suggests that, independent of weight status, BED in youth may be associated with structural brain differences in regions critical for appetite regulation. However, it remains unclear whether observed structural differences precede the development of BED. Developmentally, gray matter volume peaks in pre-adolescence and then declines though adolescence and adulthood (Giedd and Rapoport, 2010). Examining brain structure in children with LOC-eating will help elucidate neural systems associated with the subjective experience of loss of control and may help elucidate whether structural differences are cause or consequence of binging and BED.

Although it is not known if children with LOC-eating show altered brain structure, there is some initial evidence that LOC-eating may be related to alterations in brain function. Pre-adolescents (9-12-years-old) with LOC-eating and overweight showed greater activation in response to milkshake receipt in regions that support cognitive control (e.g., inferior frontal gyrus, middle frontal gyrus) and reward (e.g., caudate) compared to pre-adolescents with overweight or healthy weight but no LOC-eating (Goldschmidt et al., 2018). Additionally, 7–10-year-old children who reported LOC had reduced activation in the cerebellum in response to images of larger compared to smaller portions of food (English et al., 2019). While the cerebellum has long been known to play a role in motor control, more recent work has identified the importance of cerebellar function in satiety signaling, meal cessation, food reward, and eating-related affective processes (Low et al., 2021; Iosif et al., 2023). Further, among adolescents with overweight or obesity, those who reported LOC-eating showed lower PFC engagement during social distress/rejection compared to those without LOC-eating (Jarcho et al., 2015). Together, these studies suggest differences in neural function associated with LOC-eating that partially overlap with the pattern of structural differences seen in children and adolescents with BED.

Given that LOC-eating in children increases risk for the development of BED in adolescence, it is critically important to gain a better understanding of the neural antecedents associated with this form of disordered eating. Initial evidence suggests that BED is associated with gray matter differences in regions implicated in cognitive control, appetite regulation, and reward (Turan et al., 2021; Chen et al., 2022; Murray et al., 2022). Therefore, the current secondary analysis examined variations in brain structure between children with and without self-reported LOC eating. Estimates of brain structure were derived from gray matter volume and surface morphology (e.g., cortical thickness, gyrification). It was hypothesized that children with LOC-eating would show greater gray matter volume in regions associated with cognitive control (e.g., PFC), reward (e.g., nucleus accumbens), and interoceptive processing and satiety signaling (e.g., insula, cerebellum). This study also explored potential differences in surface morphology associated with LOC-eating in children.

## 2 Methods

### 2.1 Participants

This secondary analysis included 143 (73 female, 51%) children aged 7- to 12-years-old (Mean = 8.9, SD = 1.3) from five prior studies designed to assess child eating behavior and neural responses to food cues (English et al., 2017; Adise et al., 2018, 2019; Masterson et al., 2019; Keller et al., 2023). All studies were approved by the Institutional Review Board at The Pennsylvania State University. Exclusion criteria across the five prior studies included any parent-reported contraindications for magnetic resonance imaging (MRI) (e.g., metal implants, claustrophobia), medications that influence taste, psychological/learning disorders (e.g., attention-deficit hyperactivity disorder), food allergies, and parents reporting that the child disliked the food served in the primary studies.

### 2.2 Measures

#### 2.2.1 Demographics

Yearly family income and parental education were used as proxies for socioeconomic status (Bradley and Corwyn, 2002). The parent who accompanied the child to the visit was predominately responsible for feeding-related decisions in the home, which was the mother 87% of the time (*n* = 125).

#### 2.2.2 Anthropometrics

Child height and weight were measured twice using a stadiometer (Detecto model 437, Webb City, MO) and electronic scale (Seca model 202, Chino, CA). Body mass index (BMI; kg/m^2^) was calculated from the averaged height and weight and BMI-for-age-and-sex percentile (BMI percentile) was determined using the age and sex adjusted cut-offs from the Centers for Disease Control (Kuczmarski et al., 2002).

#### 2.2.3 LOC-eating

The Loss of Control-Eating Disorders (LOC-ED) screening form is a semi-structured interview used to assess LOC-eating (Tanofsky-Kraff et al., 2008; Altman et al., 2020) which has been shown to have both internal and external validity in pediatric samples (Goldschmidt, 2017). Children were asked: “During the past 3 months have you ever felt that you were not able to stop eating, or not able to control the type of food or amount of food that you ate?.” Children were allowed to skip or decline to answer, therefore, this sample only included children who responded as ‘yes’ or ‘no’ during the interview.

#### 2.2.4 Mock training for magnetic resonance imaging

A training session used a mock MRI scanner to familiarize children with the MRI environment and to help them practice being still. Children entered the mock scanner and were instructed to lie still while they heard sounds similar to those heard in the MRI scanner. For full protocol details, see previous functional MRI publications (English et al., 2017; Adise et al., 2018, 2019; Masterson et al., 2019).

#### 2.2.5 MRI acquisition

Motion was restricted by using padding around the head, arms, and body. While all data were acquired using the same Siemens Prisma Fit 3 T scanner, the exact parameters used for MPRAGE acquisition varied between studies (see Supplementary Table S1). Scan variability can be mitigated with inclusion of study as a covariate (Fennema-notestine et al., 2007; Pardoe et al., 2008; Chen et al., 2014; Takao et al., 2014), therefore, study number was included as a control variable in all statistical models.

### 2.3 Analytic approach

#### 2.3.1 Descriptive statistics

Participant characteristics were analyzed in R. Differences between children with and without LOC-eating were tested using χ ^2^ and Fisher exact tests for categorical variables (e.g., sex, weight status) and 2-sample *t*-tests for continuous variables (e.g., age, BMI percentile).

#### 2.3.2 MRI pre-processing

All scans were analyzed using the SPM12 software^1^ (version 7,771) and the Computational Anatomy Toolbox (CAT12^2^; version 12.8.2) in Matlab R2021b. CAT12 has been validated against FreeSurfer and does not require manual annotation of scans (Singh, 2023). A study-specific tissue probability map was generated using the CerebroMatic toolbox using age and sex as covariates (Wilke et al., 2017) because children differ anatomically from the adult-based standard tissue probability maps (Yoon et al., 2009). Scans were bias- and noise-corrected, skull stripped, and segmented into gray matter, white matter, and cerebrospinal fluid. Total intercranial volume (TIV) was computed for each child. Image quality was assessed via a weighted measure of noise and bias. While scans with a quality above 70 are considered adequate, an image quality rating (IQR) ≥ 80 is preferred (Gaser et al., 2022). Therefore, the sample used in this study had scans with IQR ≥ 80.

#### 2.3.3 Gray matter volume

Gray matter volume was extracted using CAT12’s Neuromorphometrics atlas (136 regions of interest—ROIs^3^). Additionally, to allow for better localization of sub-cortical differences (e.g., hippocampal CA1 versus CA4), gray matter volume was extracted using the Cobra atlas (Winterburn et al., 2013), which has detailed segmentation of the cerebellum and lobules, hippocampus and its subfields, the striatum, and globus pallidus (52 ROIs).

#### 2.3.4 Surface morphology

Measures of surface morphology included cortical thickness, gyrification, sulci depth, and cortical complexity. CAT12 uses projection-based thickness (Dahnke et al., 2013) to estimate cortical thickness. This process is capable of handling voxels with partial-volume and sulci blurring and asymmetries (Dahnke et al., 2013). Topological correction was performed using spherical harmonics (Yotter et al., 2011) and then a central surface mesh was created using surface refinement. Individual surfaces were spatially registered to the FreeSurfer template using spherical mapping with minimal distortions (Yotter et al., 2011). Cortical thickness surfaces were smoothed separately with a Gaussian kernel with a full width half-maximum (FWHM) of 15 mm. CAT12’s automatic calculation of cortical thickness has been shown to be valid and highly reliable with FreeSurfer estimates (Ay et al., 2022). Gyrification is the absolute mean curvature at each vertex (Luders et al., 2006) while the gyrification index reflects the local degree of cortical folding using the ratio of pial surface and surface area if no folding were present (i.e., surface ratio) (Toro et al., 2008). Sulci depth is the Euclidean distance between central surface and the convex hull which is transformed with a square root function to make it conform to a normal distribution (Yun et al., 2013). Lastly, cortical complexity is indexed by fractal dimension (Yotter et al., 2011). Surfaces for gyrification, sulci depth, and cortical complexity were smoothed using a Gaussian kernel with a FWHM of 20 mm. Surface metrics were extracted using the Desikan-Killany-Tourville atlas (Desikan et al., 2006).

#### 2.3.5 MRI analysis

Analyses of covariance (ANCOVA) was used to assess the difference in structure between those with and without LOC-eating as the model included both continuous and categorial control variables. All models adjusted for sex, age, obesity status, and study as covariates. Both obesity status and study were dummy-coded as categorical variables. For models assessing differences in gray matter volume, TIV was also included to adjust for differences in overall head size. CAT12’s whole-brain ROI approach tests the ANCOVA for each ROI in an atlas and adjusts for multiple comparisons using the Holm-Bonferroni procedure to ensure an adjusted α = 0.05. The same approach was applied to 52 ROIs from Cobra atlas (Winterburn et al., 2013), which has detailed segmentation of the cerebellum and lobules, hippocampus, hippocampal subfields, striatum, and globus pallidus.

##### 2.3.5.1 Matched samples sensitivity analyses.

As there were fewer children who reported experiencing LOC-eating (*n* = 37) than those who did not experience LOC-eating (*n* = 106; see Table 1), we identified a sub-sample of children (*n* = 37) without LOC-eating that were matched to those with LOC-eating on key demographic characteristics including age, sex, and weight status. This was done using the MatchIt package in R (Ho et al., 2011) which uses a non-parametric approach (Ho et al., 2007) based on the propensity score nearest neighbor measure (Ho et al., 2011). To confirm differences observed with the larger sample, differences in brain structure were tested between children with LOC-eating (same as full analyses) and the matched sub-sample without LOC-eating (*n* = 37) using general linear models. Demographics for the matched sub-sample are in Supplementary Table S2.

**Table 1:** Demographic characteristics.

	Total *N* = 143	LOC-eating *N* = 37	No LOC-eating *N* = 106
	Mean (SD)	Mean (SD)	Mean (SD)
Age, yr	8.9 (1.3)	8.7 (1.3)	9.0 (1.3)
BMI Percentile	55.3 (28.2)	66.3 (27.0)*	51.4 (27.8)*
BMI Z-score	0.2 (1.0)	0.6 (1.0)*	0.1 (0.9)*
TIV, ml	1,518 (118)	1,504 (120)	1,522 (117)
IQR	82.1 (1.1)	82.1 (1.3)	82.1 (1.0)
	*N* (%)	*N* (%)	*N* (%)
Sex			
Female	73 (51%)	21 (57%)	52 (49%)
Male	70 (49%)	16 (43%)	54 (51%)
Weight status			
Obesity	14 (9.8%)	6 (16%)	8 (7.5%)
Overweight	16 (11%)	5 (14%)	11 (10%)
Healthy weight	113 (79%)	26 (70%)	87 (82%)
Ethnicity			
Hispanic/Latinx	2 (1%)	1 (3%)	1 (1%)
Not hispanic/Latinx	123 (86%)	34 (92%)	89 (84%)
Unknown/PNA	18 (13%)	2 (5%)	16 (15%)
Race			
Asian	4 (3%)	0 (0%)	4 (3.8%)
Black	4 (3%)	3 (8.1%)	1 (0.9%)
White	135 (94%)	34 (92%)	101 (95%)
Mother’s education			
>BA degree	48 (34%)	11 (30%)	37 (37%)
BA degree	51 (36%)	13 (35%)	38 (38%)
<BA degree	38 (27%)	13 (35%)	25 (25%)
Unknown/PNA	6 (4%)	0 (0%)	6 (6%)
Income			
>$100,000	51 (36%)	8 (22%)	43 (41%)
$51,000–$100,000	64 (45%)	18 (49%)	46 (43%)
<$51,000	25 (17%)	10 (27%)	15 (14%)
Unknown/PNA	3 (2%)	1 (3%)	2 (2%)

BMI, body mass index; IQR, image quality rating; PNA, prefer not to answer; TIV, total intracranial volume. *p < 0.05 for difference between LOC-eating and No LOC-eating.

## 3 Results

### 3.1 Participant characteristics

Based on BMI percentile, the majority of the sample had healthy weight (BMI < 85th percentile, *n* = 113; 79%) with almost equal numbers of children with overweight (BMI 85th–94.9th percentile, *n* = 16, 11%) and obesity (BMI > 95th percentile, *n* = 14, 10%). Full participant demographics are presented in Table 1. Children with (*n* = 37) and without LOC-eating (*n* = 106) did not differ in age, sex, income, or maternal education (*ps* > 0.062). While BMI and distribution of weight status did not differ between groups (*ps* > 0.054), BMI percentile [*t*(65) = −2.86, *p* = 0.006] and BMI *Z*-score [*t*(60) = −2.90, *p* = 0.005] were higher in children who reported LOC (Table 1).

### 3.2 Structural results (Table 2)

**Table 2:** Structural differences between children with and without LOC-eating.

	H	T	Ze-value	Region
Grey Matter Volume – Neuromorphometrics Atlas				
LOC < No LOC	R	−2.12	−2.30	Parahippocampal Gyrus
LOC > No LOC	R	2.33	2.10	Orbital Frontal Gyrus (Gyrus Rectus)
Grey Matter Volume – Cobra Atlas				
LOC < No LOC	L	−2.96	−2.91	Hippocampus – CA4
	L	−1.87	−1.85	Cerebellum – Lobule IV
Sulci Depth – Desikan-Killany-Tourville Atlas				
LOC > No LOC	L	3.19	3.12	Rostral Anterior Cingulate Gyrus
	L	1.70	1.69	Cuneus
Cortical Complexity – Desikan-Killany-Tourville Atlas				
LOC > No LOC	L	3.08	3.02	Insula

LOC, loss of control; H, hemisphere; T, *t*-test statistic; Ze-value, equivalent *Z* value. Derived from analysis of covariance (ANCOVA) models adjusted for sex, age, obesity status, and study. All group differences were *p* < 0.05 after adjustment for multiple comparisons using the Holm–Bonferroni procedure. See section 2.3.5 MRI Analysis.

#### 3.2.1 Gray matter volume

**Figure 1:**
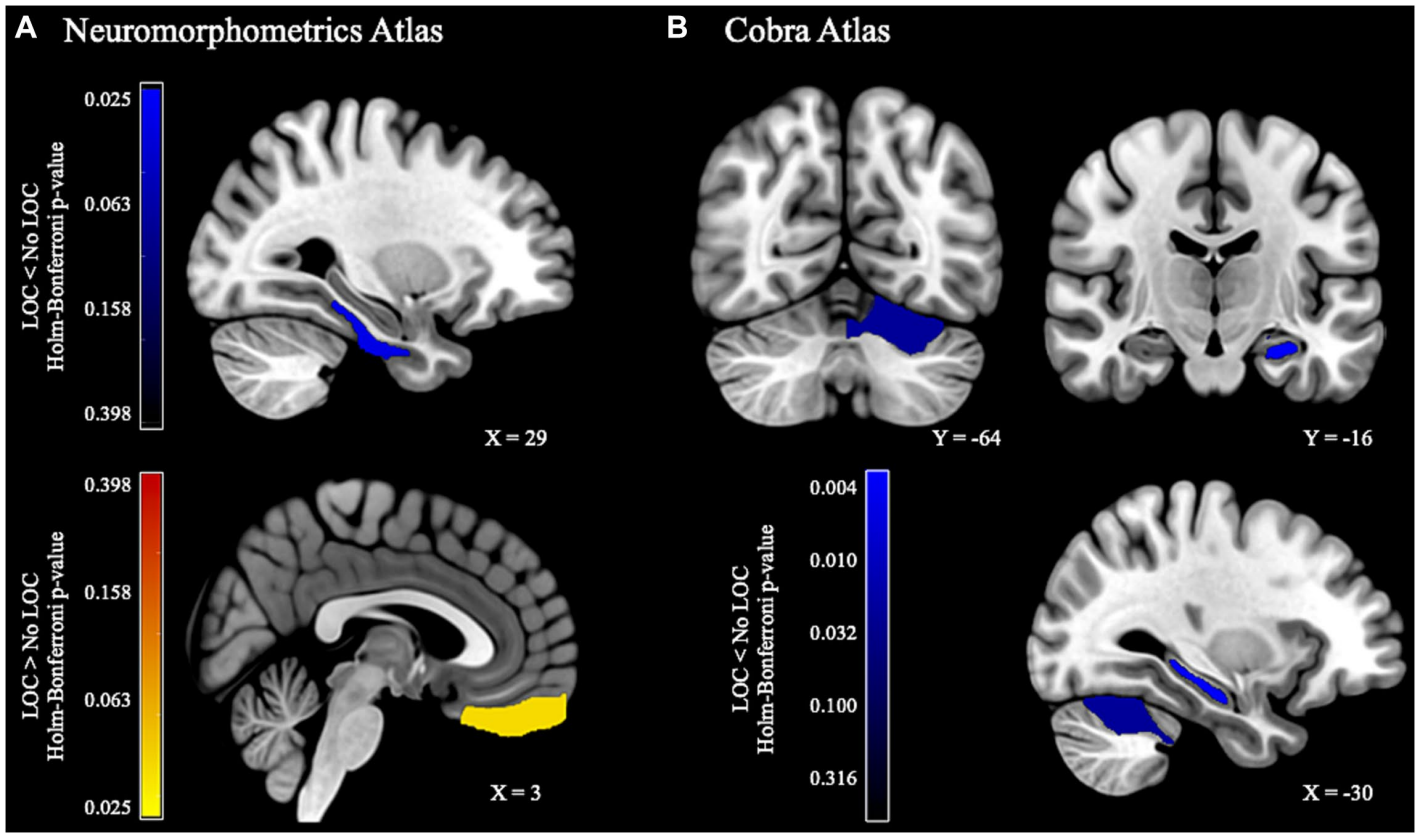
Gray matter volume differences between children with and without LOC-eating. **(A)** Whole-brain ROI-based results using the Neuromorphometrics atlas. **(B)** Subcortical results using the Cobra atlas.

Children with LOC-eating had greater gray matter volume in right gyrus rectus within orbital frontal cortex but lower gray matter volume in right parahippocampal gyrus (Figure 1A; uncorrected results in Supplementary Table S3). Additional models examining more localized sub-cortical regions found that children with LOC-eating had lower gray matter volume in left CA4/dentate gyrus subfield of the hippocampus and left cerebellar lobule VI (Figure 1B; uncorrected results in Supplementary Table S4).

##### 3.2.1.1 Matched sub-sample sensitivity test

Additional sensitivity tests were conducted to determine whether results were driven by the difference in number of children with (*n* = 37) and without LOC-eating (*n* = 106). These analyses used a sub-sample of children without LOC-eating that was matched to those with LOC-eating on key demographic variables (see 2.3.5.1 Matched Samples Sensitivity Analyses). Sensitivity tests showed consistent results as observed in the full sample apart from the gray matter differences observed in the cerebellum, potentially due to reduced power (see Supplementary materials).

#### 3.2.2 Surface morphology

**Figure 2:**
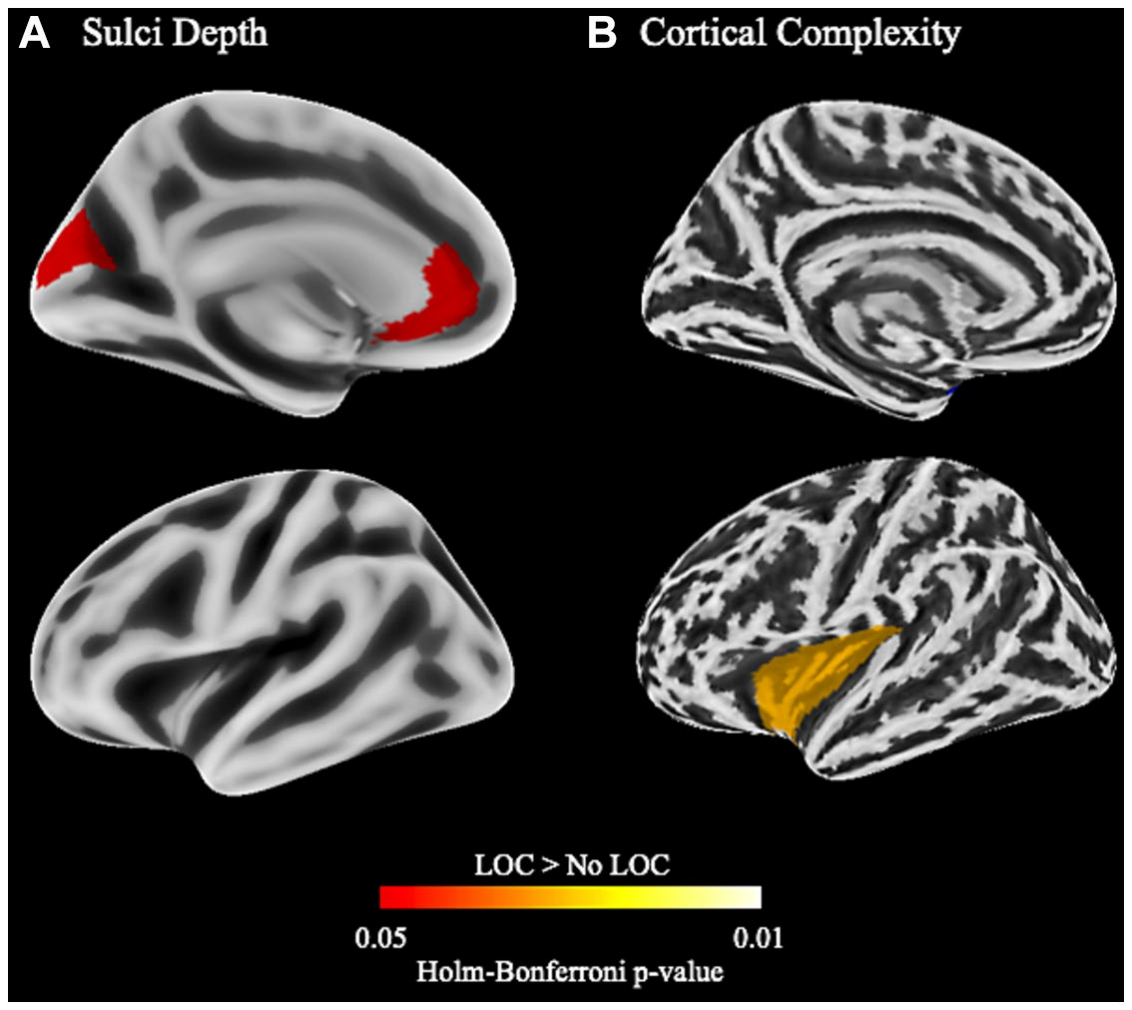
Cortical structure differences between children with and without LOC-eating using the Desikan-Killany-Tourville atlas. **(A)** Differences in sulci depth in the left hemisphere. **(B)** Differences in cortical complexity (i.e., fractal dimension) in the left hemisphere.

While there were no differences for cortical thickness, gyrification, or gyrification index, children with LOC-eating had greater sulci depth in left anterior cingulate cortex (ACC) and cuneus and greater cortical complexity in left insular cortex (Figure 2; uncorrected results in Supplementary Table S5).

##### 3.2.2.1 Matched sub-sample sensitivity test

Additional sensitivity tests were conducted using a sub-sample of children without LOC-eating that was matched to those with LOC-eating on key demographic variables (see 2.3.5.1 Matched Samples Sensitivity Analyses). Sensitivity tests with the smaller, matched sub-sample without LOC-eating showed consistent results apart from differences in sulci depth of the cuneus, likely due to reduced power (see Supplementary materials).

## 4 Discussion

To our knowledge, this is the first study to investigate differences in brain structure associated with LOC-eating in children. We showed that children with LOC-eating had lower gray matter volume in subcortical regions associated with memory and regulation of eating behavior (i.e., parahippocampal gyrus, hippocampus, and cerebellum) and greater gray matter volume in regions associated with top-down control and reward (i.e., OFC) relative to children without LOC-eating. Compared to children without LOC-eating, children with LOC-eating also had greater sulci depth in the ACC and greater cortical complexity in the insular cortex, two regions that are part of the cingulo-opercular network (Dosenbach et al., 2008). Together, these findings suggest that, prior to the development of BED, sub-clinical LOC-eating is independently associated with structural differences across a broad network of brain regions that have been associated with the regulation of eating behavior.

Similar to previous studies of BED in children (Murray et al., 2022) and adolescents (Turan et al., 2021), children with LOC-eating showed greater gray matter volume the OFC. In the current study, structural differences were localized to right gyrus rectus, which has also been associated with rapid weight gain in children in the ABCD study (Adise et al., 2022). Gyrus rectus is part of the medial OFC (Zald et al., 2014; Liu et al., 2015), which is considered to be the secondary olfactory cortex (Lundström et al., 2011) and part of the gustatory cortex (Lundström et al., 2011; Veldhuizen et al., 2011). OFC not only integrates multiple sensory modalities, but it also contributes to reward-related valuation and decision-making (Rolls et al., 2020; Seabrook and Borgland, 2020). Gyrus rectus is also considered to be part of ventral medial PFC (vmPFC), which is more broadly implicated in reward-related decision-making (Mackey and Petrides, 2014; Rolls et al., 2020). Together, this indicates that children with LOC-eating show structural differences in a region that may contribute to feeding behaviors through the integration of the rewarding properties of taste and smell with decision-making processes. That these effects were seen in children prior to the development of BED suggests that sub-clinical LOC-eating is associated with neural differences prior to the development of more serious and sustained binge eating. Although LOC-eating is a risk factor for the development of BED in adolescence, it is unclear with the observed structural alterations are related to future risk of BED. As this is the first study to show this, to our knowledge, larger, longitudinal studies are needed to replicate this finding and to examine the developmental trajectory of brain structure and eating behavior in those at risk for BED.

In addition to OFC, gray matter structural differences were observed in the medial temporal lobe (MTL). Children with LOC-eating showed lower gray matter volumes in both the parahippocampal gyrus and the CA4/dentate gyrus. Similarly, work in adults has shown that more restrained eating is associated with greater parahippocampal and hippocampal gray matter (Finch et al., 2021). The dentate gyrus supports learning contextual taste-postingestive associations (Chinnakkaruppan et al., 2014) and receives neuroendocrine (e.g., ghrelin) (Andrews, 2011) and gustatory signals (e.g., smell, taste) (Kanoski and Grill, 2017). Parahippocampal gyrus is also involved in hedonic (e.g., emotional memory) and inhibitory processes involved in feeding (Saruco and Pleger, 2021) while the hippocampus integrates external (e.g., taste, olfaction) and internal (e.g., interoceptive) signals with contextual information (e.g., place, time) (Kanoski and Grill, 2017; Kesner, 2018; Kitchigina et al., 2023). As rodent models have shown that high fat/high sugar diets and weight gain have negative impacts on hippocampal structure and function (Kanoski and Davidson, 2011), differences in eating patterns may contribute to structural differences associated with LOC-eating. Indeed, children with LOC-eating consume a greater proportion of energy from carbohydrates, specifically from snack and desert-type items (Theim et al., 2007; Tanofsky-Kraff et al., 2009a), and are more likely to snack throughout the day and in the evening (Matheson et al., 2012) compared to children without LOC-eating. Therefore, it remains unclear if structural differences in these MTL regions are due to differences in diet or contribute to the development of dysregulated eating behaviors in children with LOC-eating.

Children with LOC-eating also had lower gray matter volume in the cerebellum. The posterior cerebellar lobule VI is part of the ‘cognitive’ cerebellum and has connections with prefrontal, temporal, and cingulate regions (Siciliano et al., 2022). Lobule VI has been implicated in the motor components of feeding such as swallowing and mastication and is sensitive to circulating leptin (Iosif et al., 2023). There is also evidence from fMRI that food-cue reactivity in left lobule VI is lower in children with LOC-eating when viewing larger compared to smaller food portions (English et al., 2019). Together with evidence from animal models suggesting that lobule VI may play a role in reward prediction and expectancy (Wagner et al., 2017; Kostadinov et al., 2019), these findings suggest that lobule VI may play a role in meal cessation due to the integration of motor, satiety, and reward signals. While future studies are needed to better understand the role of cerebellum in the hedonic aspects of eating behavior, initial evidence suggests that cerebellum may play a role in LOC-eating.

In addition to gray matter volume differences, children with LOC-eating showed differences in surface morphometry. Children with LOC-eating had greater sulci depth in left ACC, which is similar to findings from the ABCD study showing altered ACC gray matter density in children with BED (Murray et al., 2022). Additionally, children with LOC-eating showed greater cortical complexity in left insular cortex. Both the ACC and insula are part of the cingulo-opercular network, which supports cognitive control (Dosenbach et al., 2008; Menon and D’Esposito, 2021). In addition to its role the cingulo-opercular network, insula is part of the gustatory cortex and supports interception and emotional regulation (Frank et al., 2013; Uddin et al., 2017; Centanni et al., 2021). Therefore, differences in cortical morphometry may impact neural systems that support cognitive control in addition to appetite and emotional regulation.

This study provided novel evidence for structural alterations in children with LOC-eating in a network of regions implicated in cognitive control, reward processing, and appetite regulation. However, these findings must be interpreted in light of some limitations. First, the present study is a secondary analysis with a limited number of participants. Additionally, only a quarter of the sample reported LOC-eating. Therefore, results need to be replicated in a study powered *a priori* to detect structural alterations associated with LOC-eating. Additionally, future studies are needed to determine whether pubertal status may moderate the impact of LOC-eating on brain structure. Also, while the current sample reflects the population in central Pennsylvania, the limited racial and ethnic diversity limits generalizability to other samples. While the sample was predominately healthy weight and models adjusted for weight status, future studies are needed to determine the relative impact of weight status and LOC-eating on brain structure. Lastly, the current sample was restricted to children without anxiety, depression, or other psychopathological conditions. Since both LOC-eating and BED are associated with elevated internalizing symptomology and psychosocial stress, it is important for future studies to examine how psychopathology and psychosocial stress may moderate the association between LOC-eating and brain development. Importantly, this study was focused on the subjective experience of LOC during an eating episode, regardless of amount consumed. Objective binge eating may also independently influence brain structure and development and risk for later BED, therefore, future studies are needed to assess both subjective and objective binge eating in children and adolescence with and without BED.

To our knowledge, this is the first study to show altered gray matter volume and surface morphology in children with LOC-eating. Children who self-reported episodes of LOC-eating had altered brain structure in a broad network of regions that support processing of taste and olfactory stimuli, motor control of chewing, visceral interception, cognitive control, and reward. This pattern of structural differences partially overlaps with previous findings in children with BED, which suggests some structural alterations may precede the development of binge eating in children. It remains unclear, however, if differences in brain structure increase risk for LOC-eating or if they are the result of dietary differences between children with and without LOC-eating. Future studies *a pirori* powered with larger sample size and the ability to measure structure longitudinally are needed to empirically test the temporal associations between brain structure and LOC-eating and BED in children. Additionally, future studies are needed to determine the role of sex and puberty in the developmental trajectory of brain structure in children with LOC-eating who develop BED compared to those who do not.

## Data availability statement

The datasets presented in this study can be found in online repositories. The names of the repository/repositories and accession number(s) can be found at: https://osf.io/dbxc6/.

## Ethics statement

The studies involving humans were approved by Institutional Review Board at The Pennsylvania State University. The studies were conducted in accordance with the local legislation and institutional requirements. Written informed consent for participation in this study was provided by the participants’ legal guardians/next of kin.

## Author contributions

AP and KK contributed to conception and design of the study, prepared the manuscript. SA, TM, and KK obtained funding for and designed the for the original studies. BF, SA, TM, LE, and NF contributed to data collection and curation for original studies. AP organized the curated the compiled database and completed statistical analysis. All authors contributed to interpretation and manuscript revision and have read and approved the submitted version.

## Footnotes

^1^https://www.fil.ion.ucl.ac.uk/spm

^2^http://www.neuro.uni-jena.de/cat/

^3^Neuromorphometrics.com
