# Supplementary material for: Loss of control eating in children is associated with altered cortical and subcortical brain structure
Source: Front Psychol. 2024 Jan 11;14:1237591. doi: 10.3389/fpsyg.2023.1237591 (PMC10808807; doi:10.3389/fpsyg.2023.1237591)
Supplement: Supplementary file 1 [file Table_1.DOCX]

Supplementary Material

Loss of Control Eating in Children is Associated with Altered Cortical and Subcortical Brain Structure

**Alaina L. Pearce^1^, Bari Fuchs^1^, Shana Adise^2^, Travis D. Masterson^1^, Nicole Fearnbach^3^, Laural English^4^, and Kathleen L. Keller^1,5^**

^1^ Department of Nutritional Science, The Pennsylvania State University, University Park, PA, USA

^2^ Division of Endocrinology, Diabetes, and Metabolism, Children's Hospital of Los Angeles, Los Angeles, CA, USA

^3^ Florida State University, Tallahassee, FL, USA

^4^ United States Department of Agriculture, Washington, DC, USA

^5^ Department of Food Science, The Pennsylvania State University, University Park, PA, USA

*** Correspondence:** Corresponding Author: [azp271@psu.edu](mailto:azp271@psu.edu)

# Sensitivity Tests – Matched Sample

When using the matched sample, the pattern of results remained the same for whole-brain grey matter volume models. Children with LOC-eating had greater grey matter volume in right orbital frontal cortex ($\beta$(se) = -0.10 (0.05), *p* = 0.036) and lower grey matter volume in the right parahippocampal gyrus ($\beta$(se) = 0.12 (0.05), *p* = 0.015) compared to the matched sample without LOC-eating. Children with LOC-eating also had lower grey matter volume in left CA4/dentate gyrus relative to the matched sample without LOC-eating ($\beta$(se) = 0.03 (0.01), *p* = 0.007), however, the difference between groups was no longer significant for the cerebellum ($\beta$(se) = 0.13 (0.15), *p* = 0.365). Similarly, while children with LOC-eating had greater sulci depth in the ACC compared to the matched sample of children without LOC-eating ($\beta$(se) = -0.12 (0.04), *p* = 0.009), the difference in the cuneus was no longer significant when using the matched sample ($\beta$(se) = 0.05 (0.05), *p* = 0.311). Lastly, the difference in cortical complexity of the left insula remained significant when using the matched sample such that children with LOC-eating showed greater complexity (i.e., fractal dimension) compared to those without LOC-eating ($\beta$(se) = -0.06 (0.02), *p* = 0.008).

# Supplementary Tables

| Table S1. Structural MRI Acquisition parameters across studies | | | |
| --- | --- | --- | --- |
| TR/TE  flip angle | Field of View  Voxel Size | Study | N |
| 1650/2.03 ms  flip angle = 9**°** | FOV = 256 mm  voxel size 1x1x1 mm | 1 | 17 |
|  |  | 2 | 20 |
|  |  | 3 | 50 |
|  |  | 4 | 40 |
| 1700/2.28 ms  flip angle = 8° | FOV = 256 mm  voxel size 1x1x1 mm | 3 | 50 |
| TR: repetition time; TE: echo time; FOV: field of view | | | |

| **Table S2. Demographic Characteristics for Matched Sample Sensitivity Tests** | | |
| --- | --- | --- |
|  | **LOC**  N = 37 | **No LOC**  N = 37 |
|  | Mean (SD) | Mean (SD) |
| Age, yr | 8.7 (1.3) | 8.6 (1.2) |
| BMI Percentile | 66.3 (27.0) | 55.2 (28.8) |
| BMI Z-score | 0.0 (0.7) | -0.3 (0.7) |
| TIV, ml | 1,504 (120) | 1,532 (111) |
| IQR | 82.1 (1.3) | 82.1 (1.1) |
|  | N (%) | N (%) |
| Sex |  |  |
| Female | 21 (57%) | 22 (59%) |
| Male | 16 (43%) | 15 (41%) |
| Weight Status |  |  |
| Obesity | 6 (16%) | 4 (11%) |
| Overweight | 5 (14%) | 4 (16%) |
| Healthy Weight | 26 (70%) | 27 (73%) |
| Ethnicity |  |  |
| Hispanic/Latinx | 1 (3%) | 0 (0%) |
| Not Hispanic/Latinx | 34 (92%) | 31 (84%) |
| Unknown/PNA | 2 (5%) | 6 (16%) |
| Race |  |  |
| Asian | 0 (0%) | 2 (5%) |
| Black | 3 (8.1%) | 0 (0%) |
| White | 34 (92%) | 35 (95%) |
| Mother's Education |  |  |
| >BA Degree | 11 (30%) | 16 (43%) |
| BA Degree | 13 (35%) | 14 (38%) |
| <BA Degree | 13 (35%) | 7 (19%) |
| Unknown/PNA | 0 (0%) | 0 (0%) |
| Income |  |  |
| >$100,000 | 8 (22%) | 16 (43%) |
| $51,000-$100,000 | 18 (49%) | 14 (38%) |
| <$51,000 | 10 (27%) | 6 (16%) |
| Unknown/PNA | 1 (3%) | 1 (3%) |
| BMI: body mass index; PNA: prefer not to answer; TIV: total intracranial volume | | |

| Table S3. Uncorrected Grey Matter Volume Differences Between Children with and without LOC-Eating using the Neuromorphometrics Atlas | | | | |
| --- | --- | --- | --- | --- |
|  | H | T | Ze-Value | Region |
| LOC > No LOC | L | 2.18 | 2.16 | Inferior Occipital Gyrus |
|  | L | 2.05 | 2.03 | Lateral Orbital Gyrus |
|  | R | 1.70 | 1.69 |  |
|  | L | 1.95 | 1.93 | Middle Occipital gyrus |
|  | R | 2.33 | 2.30 | Gyrus Rectus |
|  | L | 1.97 | 1.95 | Ocular part of the Inferior Frontal Gyrus |
|  | R | 2.36 | 2.33 | Frontal Operculum |
|  | L | 2.02 | 2.00 | Parietal Operculum |
|  | R | 2.12 | 2.10 | Central Operculum |
|  | R | 1.67 | 1.65 | Subcallosal Area |
| LOC < No LOC | L | -2.68 | -2.64 | Hippocampus |
|  | R | -1.77 | -1.76 |  |
|  | L | -2.00 | -1.98 | Parahippocampal Gyrus |
|  | R | -2.12 | -2.10 |  |
|  | R | -2.07 | -2.05 | Entorhinal Area |
|  | L | -2.32 | -2.30 | Pallidum |
|  | L | -2.07 | -2.06 | Basal Forebrain |
|  | L | -2.02 | -2.00 | Nucleus Accumbens Area |
|  | R | -2.74 | -2.70 |  |
|  | L | -1.99 | -1.97 | Caudate |
|  | R | -2.46 | -2.46 |  |
|  | L | -1.95 | -1.94 | Putamen |
|  | R | -2.51 | -2.48 |  |
|  | L | -1.73 | -1.71 | Temporal Pole |
| LOC: loss of control; H: hemisphere; T: t-test statistic; Ze-Value: equivalent Z value  Note: Derived from analysis of covariance (ANCOVA) models adjusted for sex, age, obesity status, and study. All group differences were p < 0.05, uncorrected | | | | |

| Table S4. Uncorrected Grey Matter Volume Differences Between Children with and without LOC-Eating using the Cobra Atlas | | | | |
| --- | --- | --- | --- | --- |
|  | H | T | Ze-Value | Region |
| LOC < No LOC | L | -2.97 | -2.91 | Stratum |
|  | R | -2.28 | -2.28 |  |
|  | L | -2.96 | -2.91 | CA4 |
|  | L | -2.53 | -2.49 | Globus Pallidum |
|  | L | -2.44 | -2.44 | Striatum |
|  | R | -2.90 | -2.85 |  |
|  | L | -2.43 | -2.40 | CA1 |
|  | L | -1.85 | -1.85 | Superior Posterior Lobule VI |
|  | L | -1.82 | -1.82 | Superior Posterior Lobule Crus II |
|  | R | -1.81 | -1.81 |  |
|  | L | -1.73 | -1.73 | Superior Posterior Lobule Crus I |
|  | R | -1.67 | -1.66 |  |
|  | R | -1.69 | -1.69 | Superior Posterior Lobule VIIB |
|  | R | -2.06 | -2.04 | Anterior Lobule III |
|  | R | -2.96 | -2.91 | Subiculum |
| LOC: loss of control; H: hemisphere; T: t-test statistic; Ze-Value: equivalent Z value  Note: Derived from analysis of covariance (ANCOVA) models adjusted for sex, age, obesity status, and study. All group differences were p < 0.05, uncorrected | | | | |

| Table S5. Uncorrected Cortical Differences Between Children with and without LOC-Eating using the Desikan-Killany-Tourville Atlas | | | | |
| --- | --- | --- | --- | --- |
|  | H | T | Ze-Value | Region |
| Cortical Thickness | | | | |
| LOC < No LOC | L | -1.97 | -1.90 | Pars Triangularis |
|  | L | -1.82 | -1.80 | Frontal Pole |
|  | L | -1.77 | -1.76 | Rostral Anterior Cingulate Cortex |
|  | R | -2.43 | -2.40 | Paricalcarine |
|  | R | 2.29 | -2.26 | Medial Orbitofrontal Gyrus |
|  | R | -1.78 | -1.78 | Rostral Middle Frontal Gyrus |
| Gyrification | | | | |
| LOC > No LOC | L | 2.18 | 2.15 | Frontal Pole |
|  | R | 2.31 | 2.28 | Pricalcarine |
|  | R | 1.81 | 1.80 | Transvers Temporal Gyrus |
|  | R | 1.67 | 1.66 | Pars Triangularis |
| Gyrification Index | | | | |
| LOC > No LOC | L | 1.72 | 1.71 | Entorhinal Gyrus |
|  | R | 1.86 | 1.85 | Posterior Cingulate Gyrus |
|  | R | 1.84 | 1.84 | Pars Opercularis |
| LOC < No LOC | L | -2.24 | -2.22 | Inferior Temporal Gyrus |
|  | L | -1.89 | -1.87 | Paracentral Gyrus |
|  | L | -1.74 | -1.73 | Parahippocampal Gyrus |
| Sulci Depth | | | | |
| LOC > No LOC | L | 3.19 | 3.12 | Rostral Anterior Cingulate Gyrus |
|  | R | 1.91 | 1.89 | Caudal Anterior Cingulate Gyrus |
|  | L | 2.25 | 2.25 | Rostral Middle Frontal Gyrus |
|  | L | 2.20 | 2.18 | Lingual Gyrus |
|  | L | 2.12 | 2.10 | Precuneus |
|  | L | 2.11 | 2.09 | Paracentral Gyrus |
|  | R | 2.30 | 2.27 |  |
|  | L | 1.96 | 1.94 | Pars Triangularis |
|  | R | 1.95 | 1.95 | Transverse Temporal Gyrus |
|  | R | 1.67 | 1.66 | Superior Temporal Gyrus |
|  | L | 1.88 | 1.86 | Middle Temporal Gyrus |
|  | L | 1.84 | 1.82 | Lateral Occipital Gyrus |
|  | R | 1.90 | 1.87 | Insula |
|  | L | 1.70 | 1.69 | Cuneus |
| Cortical Complexity | | | | |
| LOC > No LOC | L | 3.08 | 3.02 | Insular Cortex |
|  | L | 1.84 | 1.83 | Entorhinal Cortex |
|  | R | 1.93 | 1.91 | Precentral |
| LOC < No LOC | R | -3.06 | -3.01 | Pars Triangularis |
| LOC: loss of control; H: hemisphere; T: t-test statistic; Ze-Value: equivalent Z value  Note: Derived from analysis of covariance (ANCOVA) models adjusted for sex, age, obesity status, and study. All group differences were p < 0.05, uncorrected | | | | |
